# Analysis of Fluoride Content of Different Types of Salts Used in the Indian Diet: An In Vitro Study

**DOI:** 10.7759/cureus.66418

**Published:** 2024-08-08

**Authors:** Amritha Pai, Venkitachalam Ramanarayanan, Keerthana Rajeev

**Affiliations:** 1 Public Health Dentistry, Amrita School of Dentistry, Amrita Vishwa Vidyapeetham, Kochi, IND

**Keywords:** sources of fluoride, in vitro study, himalayan salt, fluoride concentration, salt use

## Abstract

Introduction

Salt is an essential component of the Indian diet. Edible salt contains fluoride, and its concentration varies depending on the source and manufacturing process. This study aimed to assess the fluoride concentration of commonly available varieties of edible salts in India.

Methodology

This in vitro study was conducted with four different types of edible salts viz. iodized salt, rock salt, pink salt, and black salt. Two brands of each salt available in the market were procured. Fluoride estimation was conducted using the sodium 2-(parasulphophenylazo-)- 1,8-dihydroxy-3,6-naphthalene disulphonate (SPADNS) method using Fluoride High-Range Checker® HC (Hanna Equipments India Pvt. Ltd., Mumbai, India) with HI739-26 reagent. The reaction between fluoride and the reagent forms a colorless complex in the sample. The concentration is then determined from the color produced, measured in parts per million (ppm).

Results

Iodized salt showed the least fluoride content (12.5 ± 7.5 ppm), while it was highest for black salt (77.5 ± 19.9 ppm). There was a statistically significant difference between the study groups (p=0.009). Rock salt also showed high amounts of fluoride (40.8 ± 52.4 ppm).

Conclusion

Fluoride content in different types of edible salt varied, though well within the prescribed limits. This calls for nutritional labelling of fluoride to help the consumer make informed choices.

## Introduction

Fluoride, a highly reactive element, plays a crucial role in oral health due to its chemical and physiological properties [1]. Fluoride helps the enamel remineralize [2] and also interferes with bacterial enzyme enolase activity inhibiting bacterial metabolism, thereby playing a major role in preventing dental caries. Long-term ingestion of fluoride, especially in infants and children, causes fluorosis [3]. Fluoride levels above 2 parts per million (ppm) can cause different types of fluorosis during development, such as opaque white patches, dentin hypersensitivity, mottled and pitted enamel, and severe chronic fluoride toxicity, which can damage heart cells, make ligaments hard, and cause kyphosis and changes in muscle size and shape [4].

Ingestion of fluoride is through drinking water, certain diets, salts, and inadvertent ingestion of fluoride-containing dentifrices [5]. The daily tolerable upper-level intake varies from 0.01 mg for infants to 4 mg for adults and the elderly [6], although the National Institute of Nutrition in India does not mention a recommended dietary allowance for fluoride. Groundwater in certain geographical regions of the country contains high amounts of fluoride. Dietary sources of fluoride include tea, meat, seafood, cereals, beverages, canned fruits, fluoridated milk, and drinking water [1]. The average concentration of fluoride in black tea is 3-5 ppm, whereas shellfish products contain 2-3 ppm and fruits such as grapes and raisins contain about 2.3 ppm of fluoride [7]. Dentifrices contain around 1,000-1,500 ppm of fluoride, while for children's toothpaste, it is recommended to have 1,000 ppm [8]. Certain toothpastes with zero toothpaste content are also available in the market. Food products such as semolina sourced from soil and water with high fluoride levels have a higher fluoride content [5]. Consumption of food and beverages produced using fluoridated water in non-fluoridated areas also benefits the population in the source region. The combined effect of these ingested fluorides may increase the daily recommended dietary intake, increasing the risk of fluorosis. Fluorosis is endemic in 230 districts in 19 Indian states [9], and the prevalence of dental fluorosis in India is varied and ranges from 40% to 60% in endemic regions [10].

Indian cuisine is characterized by its flavor and texture, with salt playing a crucial role in balancing sweetness and modifying texture. The rise of processed fast food and street food has led to an increase in salt consumption. Food preservation, meat and seafood curing, and cottage cheese production use salt's bacteriostatic properties as an emulsifying agent. The National Iodine Deficiency Disorders Control Programme (NIDDCP) in India adopted Universal Salt Iodization to combat iodine deficiency, which was a public health concern in the 1960s. The use of iodized salt in households decreased goiter incidence in the endemic population [11-13]. In South India, salt sources include meat, poultry, eggs, dairy, fish, seafood, and dairy products, whereas in North India, they include dairy, bread, bakery products, fruits, and vegetables [14].

Edible salt, including table salt, rock salt, pink salt, and black salt, contains fluoride concentrations varying depending on the source. Around 71% of households in India use iodized salt [11], while the NIDDCP initiative restricts the use of non-iodized salt for human consumption. Non-iodized salt is popular in North-West India for its taste, medicinal properties, and religious fasts [15]. Manufactured through a firing process, black salt emits a pungent sulfur smell and finds its application in cooking, naturopathic treatments, and vegan diets [16]. Rock salt from the sea and Himalayan region is believed to be a potent laxative and blood pressure stabilizer [17]. Himalayan pink salt is popular for its taste. Chronic fluoride toxicity is a public health concern in regions with high fluoride distribution in groundwater and soil. Reports indicate that Himalayan pink salt contains fluoride concentrations of less than 0.1 grams [18], but another study fails to mention the fluoride content of pink salt brands available in Australia [19]. Published research on black salt and its fluoride content is comparatively low, but certain organizations have recommended that children should consume black salt in moderation due to its high fluoride content [20].

Several studies have been conducted to assess the concentration of fluorides in different parts of the world. In a Mexico City study, children in a non-endemic zone for fluorosis showed overexposure to fluoride. Mexico implemented salt fluoridation in 1991 to prevent dental caries. However, in the 44 table salt brands analyzed, only three samples contained the regulated fluoride concentration, and eight samples had concentrations above the norm [21]. Similarly, literature from Colombia and Nicaragua suggests that most of the table salts available do not meet the legal requirements of fluoride content of the respective country [22,23]. Studies analyzing the effect of different types of salt and its fluoride content in India are relatively few. The nutritional information provided by the manufacturers on the labels does not contain fluorides and hampers the ability of consumers to make an informed decision about their diet and health. With the increasing use of salt in processed foods and beverages, it is critical to evaluate salt for fluorosis risk.

Thus, the objective of this study was to analyze the fluoride content of commonly available types of edible salts in India.

## Materials and methods

Study design

This was a laboratory investigation conducted at Amrita School of Dentistry.

Study sample and sample size

The study included four types of edible salt, each from two different brands, resulting in a total of eight salt samples. The types of salt examined were iodized salt (Keerthi Nirmal, Tata Salt Superlite), Himalayan pink salt (Woahganics, Brahmins), black salt (Down to Earth Morarka Organic, Natural Tattva), and rock salt (Bio Basics, Natural Tattva).

Fluoride estimation

The fluoride concentration in each salt sample was determined using Hanna Fluoride High-Range Checker® (Hanna Equipments India Pvt. Ltd., Mumbai, India) based on the sodium 2-(parasulphophenylazo-)- 1,8-dihydroxy-3,6-naphthalene disulphonate (SPADNS) method that uses a fixed wavelength light-emitting diode (LED) and silicon photo detector. The instrument detects fluoride in the range of 0 to 20 ppm with a resolution of 0.1 ppm and accuracy of ±5% of reading at 25 degrees Celsius (77 degrees Fahrenheit). Light source is a LED of 575 nm with a silicon photocell light detector.

Fluoride estimation was conducted based on the manufacturer's instructions. As the range Fluoride High Range Checker was 0-20 ppm, the salt solution was diluted in 1:10 dilution and the fluoride reading obtained was multiplied by dilution factor (x10) for the final concentration in ppm. Upon activation of the instrument, 2 mL of H1739AS reagent was added to the cuvette and filled up to 10 mL with H1739BS reagent and mixed thoroughly. The mixture was then placed into the instrument and calibrated. Following this, 1 mL of salt solution sample was added to the cuvette and mixed thoroughly. The cuvette was placed back into the meter, and concentration of fluoride displayed was recorded.

**Figure 1:**
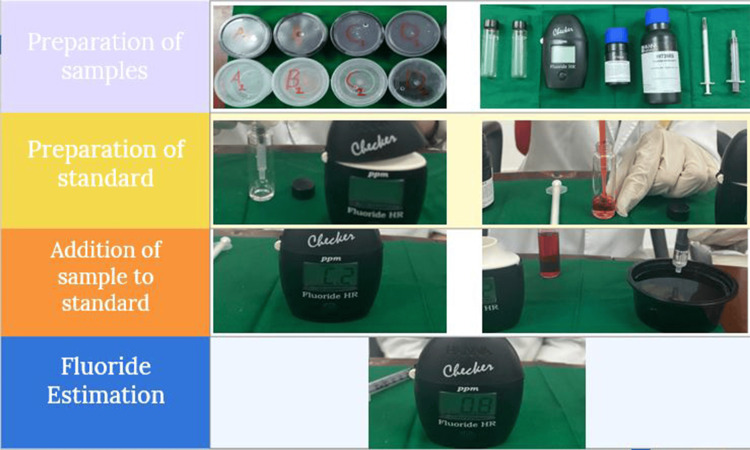
Method of fluoride estimation

Fluoride levels were measured, and the results were expressed in parts per million (ppm) (Figure 1).

Statistical analysis

The obtained results were coded, tabulated, and analyzed using Statistical Package for Social Sciences (SPSS) Version 20 (IBM Corp., Armonk, NY). Mean ppm scores of the three samples of each brand of each salt were calculated. Comparison of fluoride levels between the four salts was conducted using the Kruskal-Wallis ANOVA and post hoc comparison using the Mann-Whitney U method.

## Results

A total of 24 samples, six samples of each type of salt (iodized salt, Himalayan pink salt, Himalayan rock salt, and black salt) were analyzed. The mean fluoride levels in ppm were highest for black salt followed by rock salt, pink salt, and iodized salt.

Table 1 shows the amount of fluoride in ppm found in different types of salt. The mean fluoride concentration in iodized salt was 12.5 ± 7.5 ppm. Himalayan pink salt had a mean fluoride concentration of 20.0 ± 19.4 ppm. Himalayan rock salt had a concentration of fluoride at 40.8 ± 52.4 ppm, while black salt had the highest concentration at 77.5 ± 19.9 ppm. The Kruskal-Wallis H test indicated that there is a significant difference in the fluoride concentration between the different groups, χ2(3) = 11.48, p = 0.009, with a mean rank score of 7.5 for iodized salt, 9.58 for Himalayan pink salt, 12.58 for Himalayan rock salt, 20.33 for black salt.

**Table 1:** Comparison of fluoride concentration of different types of edible salts *The p-value is statistically significant. SD, standard deviation

Type of salt	N	Mean	SD	Median	Mean rank	p-Value
Iodized salt	6	12.5	7.5	1.25	7.50	0.009*
Himalayan pink salt	6	20.0	19.4	1.75	9.58	
Himalayan rock salt	6	40.8	52.4	2.25	12.58	
Black salt	6	77.5	19.9	8	20.33	

The post hoc Dunn's test using a Bonferroni corrected alpha of 0.0083 indicated that the mean ranks of the following pairs are significantly different: Iodized salt-black salt and Himalayan pink salt-black salt (Table 2).

**Table 2:** Post hoc pair-wise comparison of fluoride concentration of different types of edible salts *The p-value is statistically significant. X1, iodized salt; X2, Himalayan pink salt; X3, Himalayan rock salt; X4, black salt

Groups	Test statistic	Std. error	p-Value
x_1_-x_2_	-2.0833	-0.512	0.608
x_1_-x_3_	-5.0833	-1.250	0.211
x_1_-x_4_	-12.8333	-3.157	0.002*
x_2_-x_3_	-3	-0.738	0.460
x_2_-x_4_	-10.75	-2.644	0.008*
x_3_-x_4_	-7.75	-1.906	0.057

Analysis between two brands of the same salt showed no significant differences in the fluoride content except for iodized salt (Table 3).

**Table 3:** Comparison of fluoride concentration between two brands on each type of salt *The p-value is statistically significant. SD, standard deviation

Type of salt	Brand	Mean ± SD	Median	p-Value
Iodized salt	Tata Salt Superlite	6.7 ± 0.58	10.0	0.043*
	Keerthi Nirmal	18.3 ± 0.29	20.0	
Himalayan pink salt	Woahganics	6.7± 0.76	5.0	0.050
	Brahmins	33.3 ± 1.89	25.0	
Himalayan rock salt	Bio Basics	23.3 ± 1.76	25.0	0.827
	Natural Tattva	58.3 ± 7.52	20.0	
Black salt	Down To Earth	81.7 ± 0.58	85.0	0.507
	Natural Tattva	73.3 ± 3.01	70.0	

## Discussion

Every day, we ingest fluoride from various sources. Drinking water contains a fluoride concentration of 0.5-1 ppm [24]. The commonly consumed foods in our diet also have varying fluoride levels, which can be beneficial when consumed appropriately but detrimental if not managed carefully. Among dietary sources, tea stands out as one of the highest contributors to fluoride intake because it accumulates fluoride effectively [25]. A cup of tea has a fluoride concentration of 0.07 to 1.5 ppm [26]. Fruit consumption averages 0.06 ppm, while meat, fish, and poultry contribute to approximately 0.22 ppm. The average consumption of fluorides from oils and fats is 0.25 ppm, which is similar to that of dairy products. Leafy vegetables contribute to 0.27 ppm, whereas sugar and adjunct chemicals are around 0.28 ppm. Fluoride content in root vegetables is approximately 0.38 ppm, while the average consumption of grain and cereal products contributed to 0.42 ppm. Vegetables such as potatoes and legumes provide around 0.50 ppm of fluoride [6].

Salt is an essential part of the Indian diet. The variability of fluoride content in different types of edible salt is a cause for concern, with limited literature available globally on this subject. Researchers have studied the fluoride concentration in table salt to some extent, but information about other commercially marketed salt varieties is scarce. Previous research has shed light on this issue, highlighting notable variations in fluoride content among different salt brands [22,23,26-29].

As per our analysis, various types of edible salts have fluoride levels, ranging from 15.05 ppm to 62.5 ppm. For adults, the recommended daily tolerable upper intake level (UL) of fluoride is 10 mg [26]. This surpasses the maximum permissible limits of fluoride consumption. Salt, which is an inevitable component of our daily diet, when used in conjunction with other food sources can adversely affect adult human health. Long-term ingestion of small amounts of fluoride results in chronic fluoride toxicity, which has a significant impact on dental and skeletal health. Most of us are unaware of this daily excess of fluoride consumption and its potential health risks. Therefore, we should meticulously regulate fluoride intake from various dietary sources to minimize foreseeable health hazards.

Brazil conducted a study that evaluated 11 brands of table salt, of which nine had fluoride. The fluoride concentrations ranged from below the Nicaraguan legislation's prescribed limit of 200-225 mg F/kg to within it. Two fluoridated brands met the legal requirements, while the remaining five fell below the specified range. Additionally, traces of fluoride were observed in two non-fluoridated brands, pointing out discrepancies in labelling or manufacturing processes [22]. In our study, none of the salts had a mention of fluoride in the Nutrition Facts labelling the packaging of the salt. This probably could be due to the absence of any regulation that mandates its display in India.

Similarly, in Bogota, Colombia, a study analyzed 28 brands of table salt and found a mean fluoride concentration of 133.8 ppm F, with values ranging widely from 4.8 to 225.7 ppm F. Notably, the majority of samples (71%) had fluoride levels below those recommended by the national program for salt fluoridation in Colombia, indicating potential inadequacies in the fluoridation process or variability in product formulation [23]. In four municipalities of Colombia, another study revealed that the average fluoride concentration of table salt fell below the lower limit (180-220 μg/g) set by the Colombian regulation [27].

Furthermore, a study examining 44 table salt brands in Mexico found significant discrepancies between the labelled fluoride content and actual concentrations. While the Mexican regulation stipulates a fluoride range of 200-250 ppm F, only a few samples fell within this range, with the majority either below or above it. Alarmingly, the salt industry needs stricter quality control measures and accurate labelling practices as 92% of the samples had incorrect fluoride content labels [28].

A pilot study in Lima, Peru, evaluated the fluoride concentration in salt packages marketed using a standardized protocol. The results showed that the mean fluoride concentration in four packages was in line with the Peruvian regulation, but in three packages it was lower. We observed variability in fluoride concentration within and among brands, ranging from 72.0 to 1,449.7 mg F/kg [29].

In our study, the fluoride levels in edible salts were well within the prescribed limits for fluoride intake. However, black salt, commonly used in Northern India, Pakistan, and Bangladesh, had a comparatively high concentration of fluoride. There is a lack of scientific literature to validate the observations, though some sources have pointed out increased fluoride concentrations in this type of salt [30-32]. Himalayan pink salt had low levels of fluoride. Grey literature sources, which mention traces of fluoride in pink salts [18,33], also corroborate this finding.

This study adds to a relatively unexplored area of fluoride concentrations in one of the commonest ingredients used in the Indian diet and also sheds light on the need for fluoride labelling in food products.

Limitations

This study, which was exploratory in nature, included only two brands of salt from each type of salt. To account for any variability, further research could include more brands and increase samples. Fluoride estimation was conducted using spectrophotometric methods using laboratory-based devices. Fluoride levels estimated using ion-selective electrodes and ion chromatography could yield more accurate results.

## Conclusions

Fluoride content in different types of edible salt varied significantly. The mean fluoride levels were highest in black salt, followed by rock salt, pink salt, and iodized salt. A statistically significant difference was found in fluoride concentration between the different types of salt. Differences in concentration were more significant between iodized salt-black salt and Himalayan pink salt-black salt pairs. No significant differences were found between two brands of the same salt, except for iodized salt, suggesting that fluoride concentration depends on the source. However, the concentration of fluoride was well within the prescribed limits required to cause fluorosis. This research emphasizes the need for understanding the fluoride concentration in various salt varieties to mitigate health hazards. It also emphasizes the need to monitor fluoride levels in edible salts and establish standardized guidelines to guarantee customer safety and promote oral health advantages.
